# Hallucination Rates and Reference Accuracy of ChatGPT and Bard for Systematic Reviews: Comparative Analysis

**DOI:** 10.2196/53164

**Published:** 2024-05-22

**Authors:** Mikaël Chelli, Jules Descamps, Vincent Lavoué, Christophe Trojani, Michel Azar, Marcel Deckert, Jean-Luc Raynier, Gilles Clowez, Pascal Boileau, Caroline Ruetsch-Chelli

**Affiliations:** 1 Institute for Sports and Reconstructive Bone and Joint Surgery Groupe Kantys Nice France; 2 Orthopedic and Traumatology Unit Hospital Lariboisière Assistance Publique-Hôpitaux de Paris Paris France; 3 Université Côte d’Azur, INSERM, C3M, Team Microenvironment, Signalling and Cancer Nice France

**Keywords:** artificial intelligence, large language models, ChatGPT, Bard, rotator cuff, systematic reviews, literature search, hallucinated, human conducted

## Abstract

**Background:**

Large language models (LLMs) have raised both interest and concern in the academic community. They offer the potential for automating literature search and synthesis for systematic reviews but raise concerns regarding their reliability, as the tendency to generate unsupported (hallucinated) content persist.

**Objective:**

The aim of the study is to assess the performance of LLMs such as ChatGPT and Bard (subsequently rebranded Gemini) to produce references in the context of scientific writing.

**Methods:**

The performance of ChatGPT and Bard in replicating the results of human-conducted systematic reviews was assessed. Using systematic reviews pertaining to shoulder rotator cuff pathology, these LLMs were tested by providing the same inclusion criteria and comparing the results with original systematic review references, serving as gold standards. The study used 3 key performance metrics: recall, precision, and *F*_1_-score, alongside the hallucination rate. Papers were considered “hallucinated” if any 2 of the following information were wrong: title, first author, or year of publication.

**Results:**

In total, 11 systematic reviews across 4 fields yielded 33 prompts to LLMs (3 LLMs×11 reviews), with 471 references analyzed. Precision rates for GPT-3.5, GPT-4, and Bard were 9.4% (13/139), 13.4% (16/119), and 0% (0/104) respectively (*P*<.001). Recall rates were 11.9% (13/109) for GPT-3.5 and 13.7% (15/109) for GPT-4, with Bard failing to retrieve any relevant papers (*P*<.001). Hallucination rates stood at 39.6% (55/139) for GPT-3.5, 28.6% (34/119) for GPT-4, and 91.4% (95/104) for Bard (*P*<.001). Further analysis of nonhallucinated papers retrieved by GPT models revealed significant differences in identifying various criteria, such as randomized studies, participant criteria, and intervention criteria. The study also noted the geographical and open-access biases in the papers retrieved by the LLMs.

**Conclusions:**

Given their current performance, it is not recommended for LLMs to be deployed as the primary or exclusive tool for conducting systematic reviews. Any references generated by such models warrant thorough validation by researchers. The high occurrence of hallucinations in LLMs highlights the necessity for refining their training and functionality before confidently using them for rigorous academic purposes.

## Introduction

The advent of artificial intelligence (AI) has led to significant advancements in various fields, including medical research. Large language models (LLMs), such as ChatGPT (OpenAI), could assist academic researchers in a variety of tasks, including writing scientific papers. These models have the potential to streamline the way researchers conduct literature searches, synthesize findings, and draft systematic reviews [1]. However, there is ongoing debate surrounding their reliability, ethical considerations, and appropriate use in academic publishing.

Recently, editorials and opinion papers have been published addressing the use of LLMs in the scientific community. One such example is an editorial in *The Lancet Digital Health*, which discusses the potential benefits and challenges of implementing AI in medical research [2]. As the application of LLMs such as ChatGPT in research settings grows, concerns have arisen regarding their accuracy, the potential for generating misleading or false information, and the ethical implications of using AI-generated content without proper disclosure.

While it is known that ChatGPT can help researchers write papers [3-5], controversy exists about whether it should be used at all, whether its use should be disclosed, and whether it should be listed as an author or not [6]. These debates raise important questions about the role of AI in scientific research and the potential consequences of using LLMs in generating systematic reviews and other research outputs [7].

In this study, we aim to address these concerns by systematically evaluating the reliability of ChatGPT and Bard (subsequently rebranded Gemini; Google AI) [8] in the context of searching for and synthesizing peer-reviewed literature for systematic reviews. We will compare their performance to that of traditional methods used by researchers, investigate the extent of the “hallucination” phenomenon, and discuss potential ethical and practical considerations for using ChatGPT and Bard in academic publishing. By providing evidence-based insights into the capabilities and limitations of LLMs in medical research, we hope to contribute to the ongoing debate about the role of AI in the research ecosystem and guide researchers in making informed decisions about using LLMs in their work.

## Methods

### Ethical Considerations

Ethics approval is not required, as human participants were not involved in this research. Consent for publication has been provided from all identifiable persons in the figures.

### Study Design

This study follows a sequential design, chosen for its ability to progressively build on each preceding phase, thus ensuring a comprehensive evaluation of the LLMs in the context of a systematic review. The process initiated with a systematic review search on PubMed, followed by the retrieval of selected papers. Subsequently, the methodology of these papers served as inputs to the LLM, which is tasked to search for papers using the same inclusion criteria as the systematic reviews. The final phase involves a comparison of the LLM results with the systematic review references, which act as the ground truth, thus providing a robust evaluation of the LLMs’ ability to replicate the results of human-conducted systematic reviews. The ethical considerations of using AI, specifically LLMs, in research were carefully evaluated.

### Systematic Review Search on PubMed

On July 27, 2023, a literature search was performed on PubMed to find literature published in the English language during 2020. The selected year aligns with ChatGPT’s training cut-off point in September 2021, ensuring that the AI model has access to the comprehensive scope of literature for the given year. The focus was directed toward systematic reviews of randomized clinical trials pertaining to shoulder rotator cuff pathology. This prevalent condition spans multiple disciplines inclusive of surgery, anesthesiology, sports medicine, and physical therapy, thereby positioning it as an optimal candidate for this multidisciplinary appraisal. In addition, the collective clinical and scientific experience of the research team on the topic furnished a critical review of the references obtained from the PubMed search and the LLMs [9-12].

An electronic search of PubMed was conducted using a combination of keywords, including “shoulder,” “rotator cuff,” and “randomized” (Multimedia Appendix 1). The search was restricted to papers published in 2020 and filtered to retrieve only systematic reviews and meta-analyses. Titles and abstracts were scrutinized, and papers indicating a systematic review of randomized studies on rotator cuff pathology were selected for further analysis.

Exclusion criteria were applied to eliminate papers that did not meet our study focus. Papers were excluded if they were not systematic reviews, if their primary concern did not pertain to rotator cuff pathology, if written in a language other than English, or if they included nonrandomized clinical studies.

Two independent reviewers (MC and PB) screened titles, abstracts, and full texts retrieved by this query. Differences between reviewers were reconciled with a third reviewer (JD). To ensure the selection of relevant systematic reviews, the reviewers applied exclusion criteria that consisted of systematic reviews including nonrandomized studies and papers that were not systematic reviews. The eligibility of the selected systematic reviews was further validated by assessing their adherence to the PRISMA (Preferred Reporting Items for Systematic Reviews and Meta-Analyses) guidelines [13]. Additionally, the registration status of these reviews was verified in the PROSPERO database [14].

For each paper referenced in the systematic reviews, information on the paper title, author list, country (based on the first author’s affiliation with PubMed), journal name, journal date and issue, DOI, and open access status was collected. We assessed the hypothesis that LLMs may favor publicly available papers in their results by using a broad definition of “open access.” This definition included open access through the journal or any full-text PDF available on another server and accessible through a Google search (eg, ResearchGate or university website).

### Systematic Review on LLMs: Paper Retrieval

**Figure 1:**
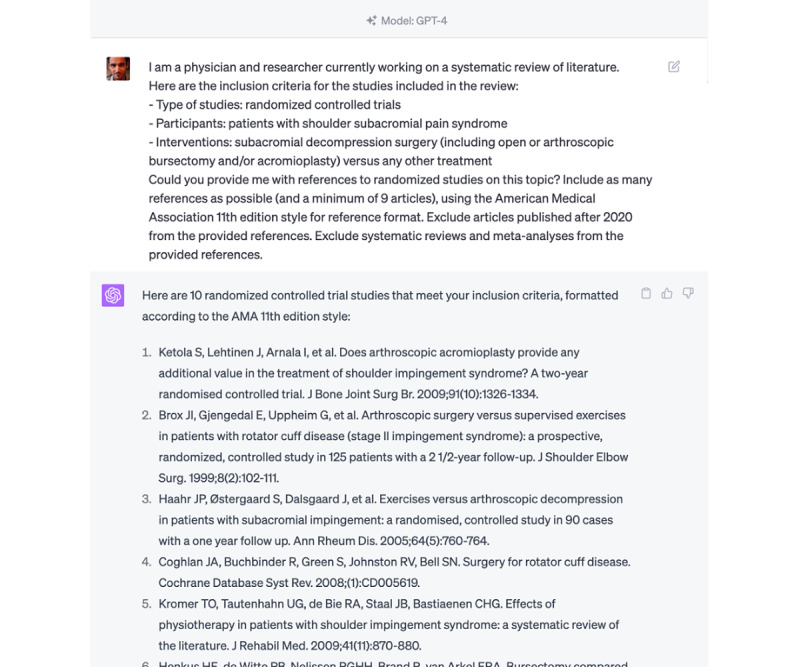
Captured screenshots demonstrating a prompt to large language models.

For each new request, a fresh chatbot session was initiated to prevent any carryover effect from previous queries, ensuring the validity of the results. We prompted ChatGPT and Bard with a precise query to identify papers that could be included in the systematic review. The structure of the prompt consisted of a statement about the physician’s and researcher’s current work, followed by the inclusion criteria for the studies in the review (Figure 1). The criteria specified randomized controlled trials with specific participant criteria and interventions comparing 2 different treatments. LLMs were asked to provide references to randomized studies on the topic, excluding papers published after 2020 and systematic reviews or meta-analyses. To assess the impact of the prompt’s specificity on the search results of LLMs, we tested 2 versions of the prompt for each request. One specifying the minimum number of papers to be found and the other without specifying this minimum number, thus providing us with an opportunity to assess if the presence or absence of a target number influences the LLMs’ search results. The query that led to the largest number of results was retained for this study.

For each paper provided by LLMs, information on the existence or hallucination status of the paper, authors’ list, country (based on the first author’s affiliation on PubMed), open-access status, inclusion in the original systematic review, randomization status, participant criteria adherence, intervention criteria adherence, exclusion of systematic reviews (as requested in the prompt), and accuracy of the provided information (authors’ list, journal, year and issue, title, and DOI) was collected. We also verified if the paper was published before 2021, as requested in the prompt.

Papers were considered hallucinated if any 2 of the following information were wrong: title, first author, or year of publication. The hallucination rate was calculated to quantify the proportion of LLM-generated references that were irrelevant, incorrect, or unsupported by the available literature, offering insights into the extent of spurious or inaccurate information production by the LLMs.

For noncomparative studies, the intervention criteria were considered adequate if at least 1 of the 2 interventions was studied in the proposed reference. For comparative studies, the intervention criteria were considered adequate if both interventions were studied in the proposed reference.

### Comparison of LLMs Results

**Figure 2:**
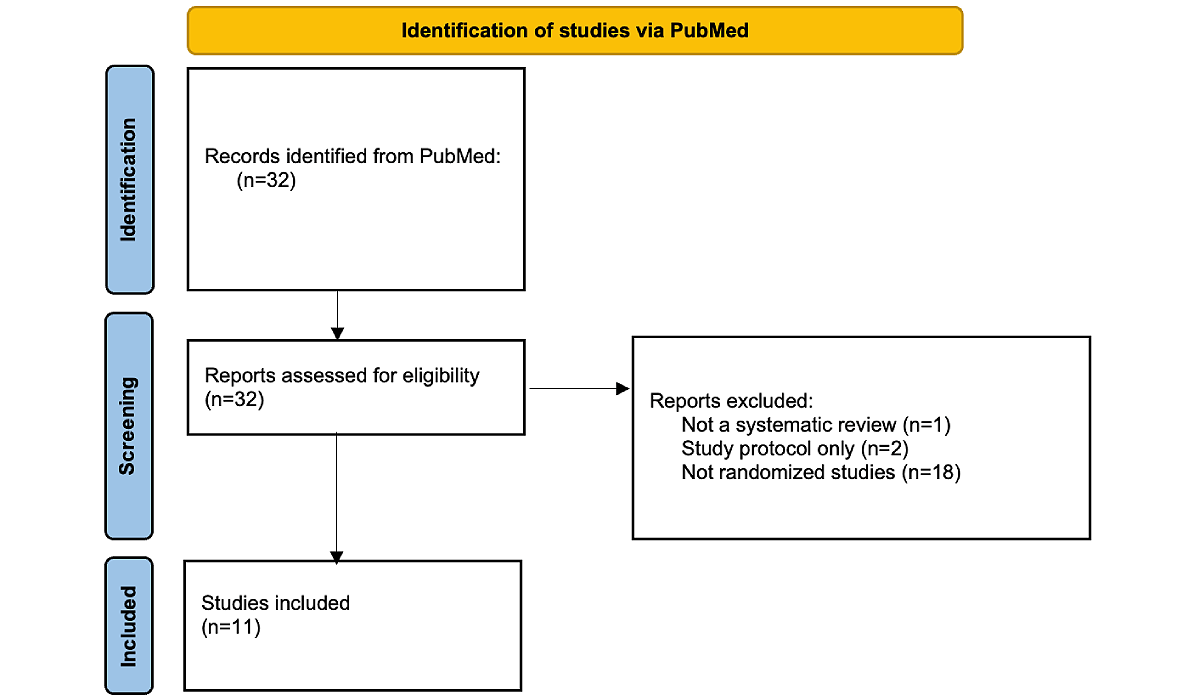
Flow diagram of included systematic reviews.

The sample size was determined based on an anticipated 10% rate of systematic review references overlooked by LLMs, with an assumed power of 90% and an α of .05. This calculation yielded a requisite of 80 references for the comparison. The PubMed search yielded 11 systematic reviews (Figure 2), each with an average of 9.9 (SD 6.6; range 3-23) references. The evaluation of the LLMs was predicated on three widely used metrics: (1) recall, representing the proportion of genuinely pertinent papers from the original systematic reviews accurately identified and retrieved by the LLMs; (2) precision, quantifying the proportion of papers retrieved by the LLMs that are verifiably present in the original systematic reviews; and (3) *F*_1_-score, which serves as an aggregate metric encapsulating both the recall and precision values (Table 1).

**Table 1:** Recall, precision, and F1-score.

	Papers provided as an output by LLMs^a^	Papers not provided as an output by LLMs
Papers cited by systematic reviews	True positive	False negative
Papers not cited by systematic reviews	False positive	True negative

^a^LLM: large language model.



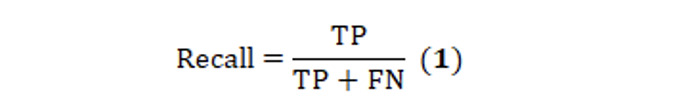





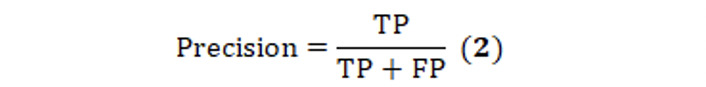





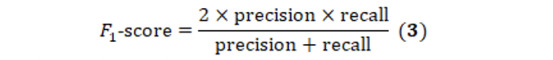



where TP represents true positive, FN represents false negative, and FP represents false positive.

The LLMs incorporated in this study included GPT-3.5 (text-davinci-002-render-sha, July 19 version; OpenAI), GPT-4 (gpt-4-32k-0314, July 19 version; OpenAI) [15], and Bard (PaLM version 2.0, released on July 13, 2023; Google AI). We conducted chi-square tests to compare each piece of information extracted from LLMs’ responses, including authors’ nationalities and the open-access status of the retrieved papers. The significance threshold used was *P*<.05. Statistical analysis was performed with EasyMedStat (version 3.24).

## Results

In total, 11 systematic reviews were identified in 4 fields (Table 2): physiotherapy (3 papers), sports medicine (3 papers), orthopedic surgery (3 papers), and anesthesiology (2 papers), leading to 33 prompts to LLMs (3 tested LLMs×11 systematic reviews). LLM prompts returned references in 32 of 33 cases: Bard did not return any result for the systematic review about “subacromial analgesia via continuous infusion catheter.” In most cases, the number of references returned by LLMs was greater or equal to that of the original papers (Table 2). Overall, 471 references were included in this study and analyzed.

Papers identified by LLMs were present in the original systematic reviews (precision) in 9.4% (13/139), 13.4% (16/119), and 0% (0/104) of cases for GPT-3.5, GPT-4, and Bard (*P*<.001), respectively. Conversely, 11.9% (13/109) of papers from the systematic reviews (recall) were retrieved by GPT-3.5, and 13.7% (15/109) by GPT-4. No paper from the systematic reviews was retrieved by Bard (*P*<.001; Table 3)*.*

**Figure 3:**
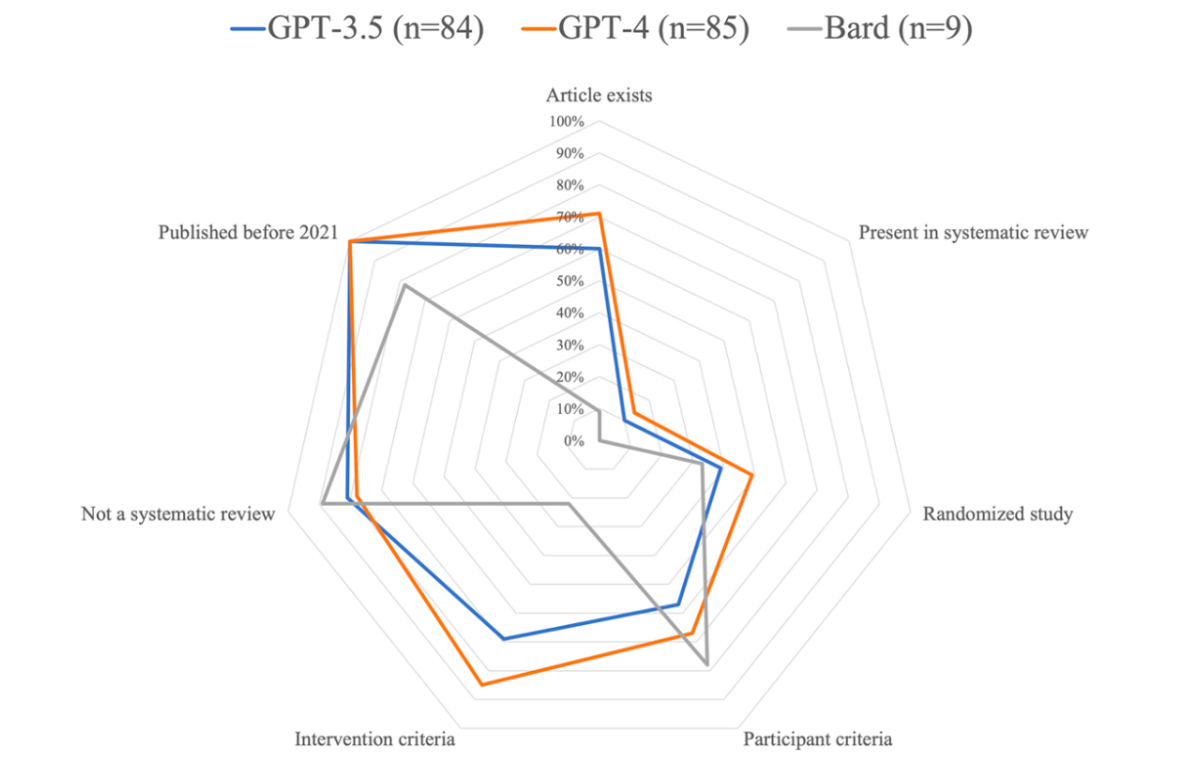
Efficiency of the tested large language models in complying to inclusion and exclusion criteria. With the exception of the “paper exists” criteria, hallucinated papers were excluded from this analysis.

The hallucination rates were, respectively, 39.6% (55/139), 28.6% (34/119), and 91.4% (95/104) for GPT-3.5, GPT-4, and Bard (*P*<.001). When analyzing the papers retrieved by GPT that were not hallucinated (n=84 for GPT-3.5 and n=85 for GPT-4), the following criteria were successfully identified (Figure 3): randomized studies (33/84, 39% vs 42/85, 49%; *P*=.24), participant criteria (49/84, 57% vs 57/85, 67%; *P*=.24), intervention criteria (58/84, 69% vs 72/85, 85%; *P*=.03), not a systematic review (69/84, 81% vs 66/85, 78%; *P*=.73), and published before 2021 (84/84, 100% vs 85/85, 100%; *P>*.99). In total, 9 papers retrieved by Bard were not hallucinated. This limited sample was not appropriate for further inferential statistics.

**Figure 4:**
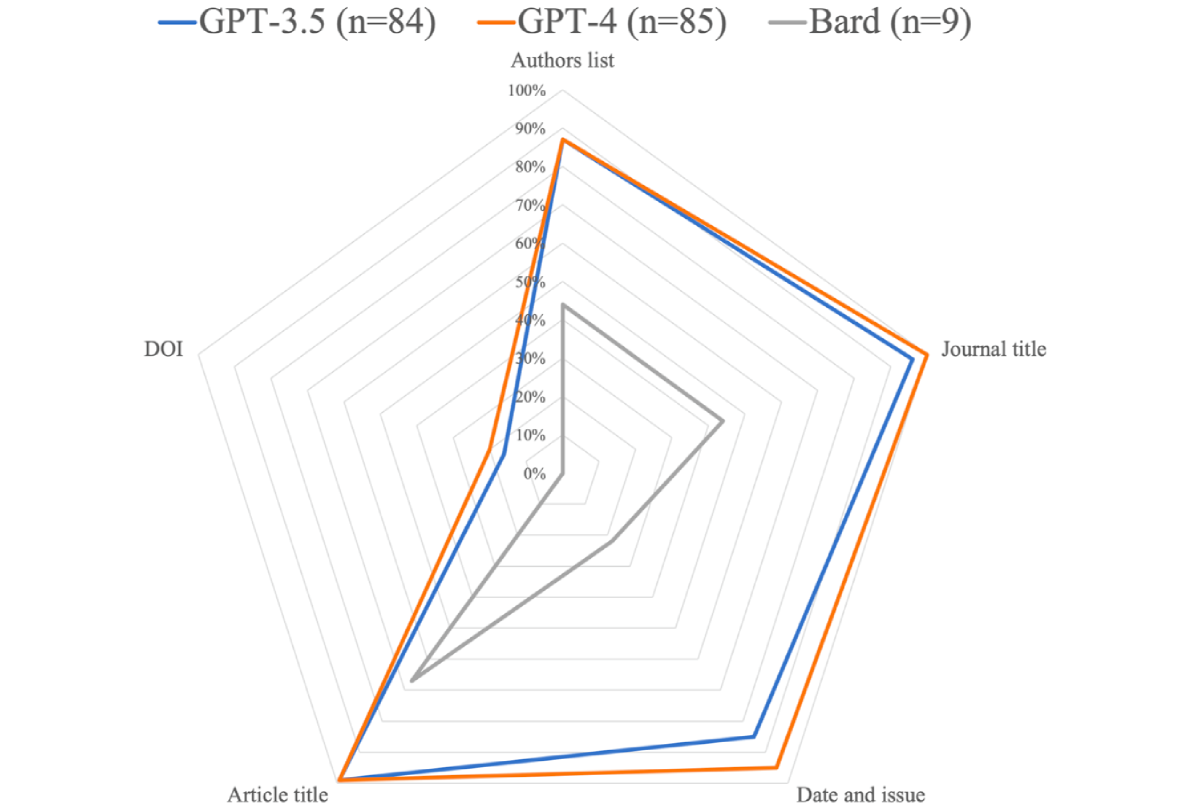
Efficiency of the tested large language models in generating accurate bibliographic information of the retrieved papers. Hallucinated papers were excluded from this analysis.

Regarding the same nonhallucinated papers retrieved by GPT, the following bibliographic information were considered accurate (Figure 4): authors list (73/84, 87% vs 74/85, 87%; *P>*.99), journal title (81/84, 96% vs 85/85, 100%; *P*=.12), date and issue (71/84, 84% vs 81/85, 95%; *P*=.02), paper title (83/84, 99% vs 84/85, 99%; *P>*.99), and DOI (13/82, 16% vs 17/84, 20%; *P*=.59).

Open-access papers were selected in 27.5% (30/109) of original systematic reviews, 38% (32/84) of GPT-3.5 papers, and 36% (31/85) of GPT-4 papers (*P*=.24). Papers from American authors were selected in 16.5% (18/109) of original systematic reviews, 44% (37/84) of GPT-3.5 papers, and 33% (28/85) of GPT-4 papers (*P*<.001).

**Table 2:** Systematic reviews included in the study and the count of papers retrieved by original authors and large language models.

Systematic review	Field	PRISMA^a^ guidelines	PROSPERO registration	Papers in the original paper, n	Papers returned by GPT-3.5, n	Papers returned by GPT-4, n	Papers returned by Bard, n
Lähdeoja et al [16]	Surgery	Yes	Yes	9	10	10	9
Catapano et al [17]	Sports medicine	Yes	No	5	7	5	5
Gutiérrez-Espinoza et al [18]	Physiotherapy	Yes	Yes	7	15	7	7
Chen et al [19]	Sports medicine	Yes	No	18	18	18	16
An et al [20]	Anesthesiology	Yes	Yes	9	9	9	0
Craig et al [21]	Surgery	Yes	No	23	22	22	23
Naunton et al [22]	Physiotherapy	Yes	Yes	7	10	8	7
Malliaras et al [23]	Physiotherapy	Yes	Yes	3	5	7	5
Simpson et al [24]	Sports medicine	Yes	Yes	18	18	18	23
Belk et al [25]	Anesthesiology	Yes	No	5	10	8	5
Belk et al [8]	Surgery	Yes	No	5	15	7	5
Total		11/11	6/11	109	139	119	104

^a^PRISMA: Preferred Reporting Items for Systematic Reviews and Meta-Analyses.

**Table 3:** Evaluative metrics of the assessed large language models.

Metric	GPT-3.5	GPT-4	Bard
True positive	13	16	0
False positive	126	103	104
False negative	96	93	109
Recall (%)	11.9	13.7	0
Precision (%)	9.4	13.4	0
*F*_1_-score (%)	10.5	14	0

## Discussion

### Principal Findings

The most important finding of this study is that using LLMs such as ChatGPT and Bard to conduct systematic reviews for a common condition such as rotator cuff disease can generate misleading or “hallucinated” references, exceeding a 25% rate.

This concern has been broached in previous literature [26-29], but our study provides an experimental design to probe the matter more deeply. OpenAI, the developer of ChatGPT, acknowledges this issue, stating that their model “occasionally generates plausible but incorrect or nonsensical responses” [30]. As LLMs increasingly assist academic researchers in producing scientific literature, this phenomenon warrants careful scrutiny.

**Figure 5:**
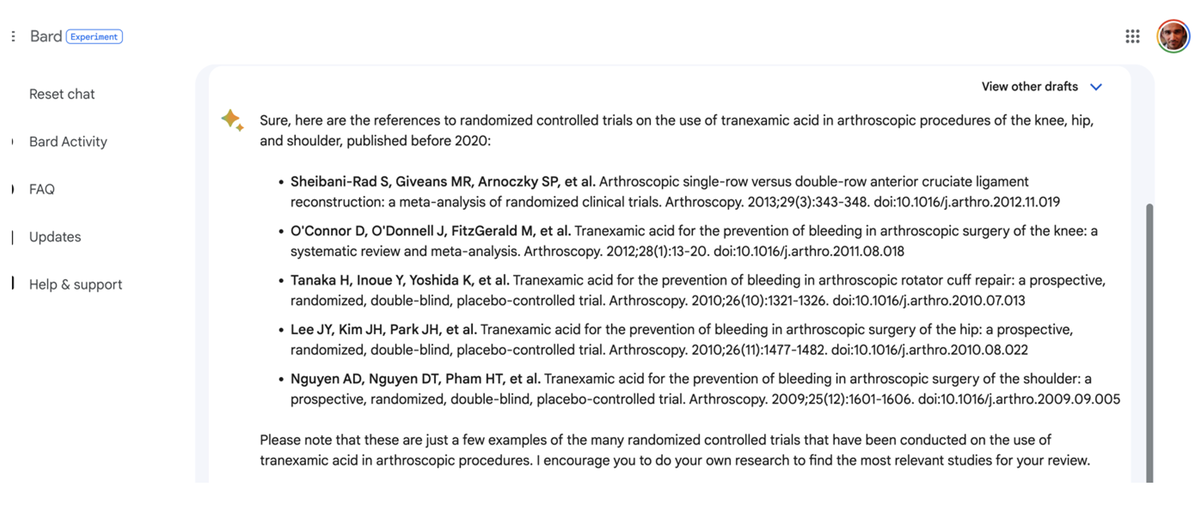
Instances of hallucinated papers. In total, 4 of 5 paper titles commence with “Tranexamic acid for the prevention of bleeding in arthroscopic” and were allegedly published in the journal Arthroscopy.

When comparing the 3 models tested, GPT-4 was the most efficient to retrieve nonhallucinated references, while GPT-3.5 produced 39.6% (55/139) of nonexisting references. Bard, however, appears ill-suited for conducting systematic reviews in the selected areas, with 91.3% (95/104) of the references failing to correlate with legitimate papers. Bard seemed to have a try-and-repeat approach, providing multiple versions of hallucinated papers with close titles and journal names (Figure 5).

**Figure 6:**
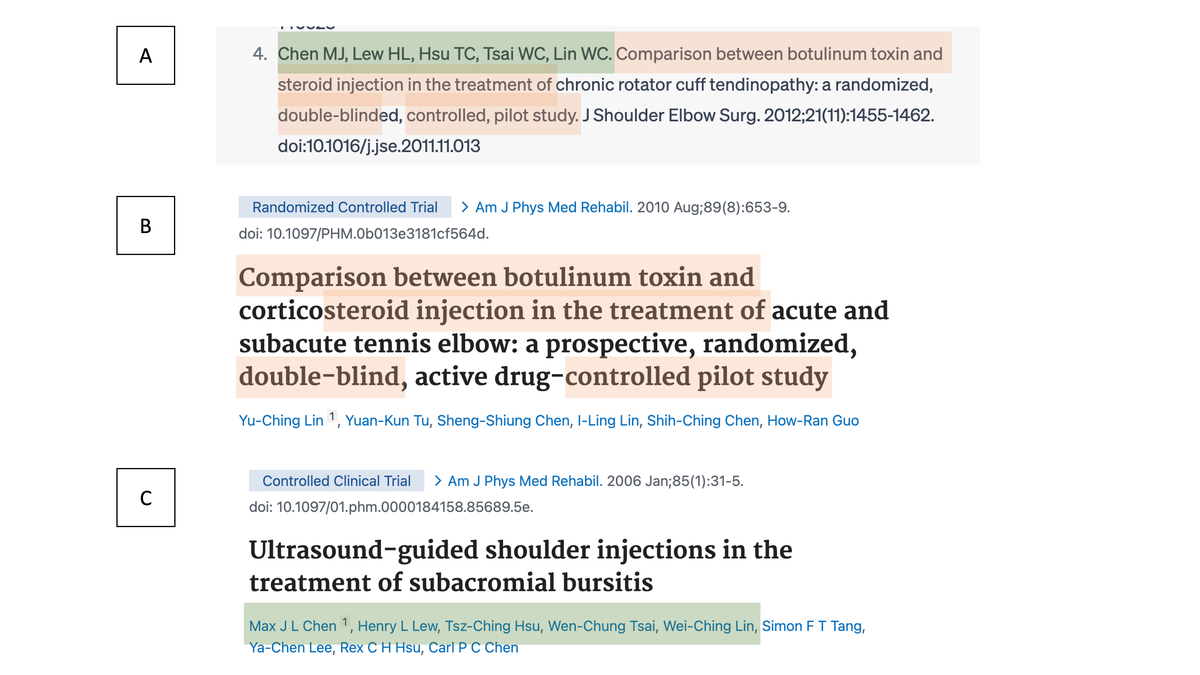
Instance of a hallucinated reference. (A) The output of a large language model. (B and C) Authentic papers with similarities in title and author list, potentially serving as original data for large language model reference generation.

Despite this, LLMs typically encouraged users to conduct their own systematic reviews, recognizing the necessity of human involvement. However, in none of our queries did the LLMs ask to verify the authenticity of the produced citations. Nonetheless, the convincing verisimilitude of the references generated by LLMs presents a risk for incautious researchers, potentially undermining the quality of scientific bibliographies if improperly used (Figures 5 and 6). Moreover, the efficiency of LLMs in retrieving original papers from systematic reviews ranged from negligible to modest (0/109, 0% to 15/109, 13.8%), emphasizing that researchers should not overly rely on these tools for systematic reviews. Nevertheless, in numerous instances, both ChatGPT and Bard “encouraged [users] to conduct their own research” (Figure 5), a suggestion that appears crucial considering the findings of this study.

It could be expected that LLMs were not able to retrieve the same references as authors of systematic reviews. However, this study also reveals that LLMs, despite being provided with the same eligibility criteria as those in the original systematic reviews, were not able to consistently apply them. For instance, the criterion of “randomized study” was adhered to in only 39% (33/84) to 49% (42/85) of nonhallucinated papers generated by ChatGPT, even when the term “randomized” appeared in the title or abstract of the papers from the original systematic reviews. The same finding was observed for the “not a systematic review” criterion, which was not respected in 20.1% (36/179) of cases, while the publicly available information of the produced papers clearly states the nature of these studies.

These discrepancies could potentially stem from the underlying statistical nature of these LLMs, which predict subsequent text (tokens) based on a model reinforced by human feedback [31]. However, as human supervision does not extend to validating the accuracy of LLM outputs, especially in specialized fields like medicine, inaccuracies can prevail.

In the case of nonhallucinated papers, however, ChatGPT demonstrated significant efficiency in retrieving accurate bibliographic information like the exact paper title, the authors’ list, and the journal title.

Potential biases in LLMs due to training on biased data sets and the risk of perpetuating stereotypes have been highlighted [2]. Our findings suggest that American authors were more frequently represented in ChatGPT references. However, further investigation across diverse medical fields is warranted to ascertain whether these LLMs may introduce such biases definitively.

### Strengths and Limitations

This investigation, by virtue of its specific and circumscribed parameters, comes with several inherent limitations. The scope of the study was exclusively focused on systematic reviews related to shoulder rotator cuff pathology. Consequently, it must be recognized that the findings might not be universally applicable across diverse medical specialties or disciplines. The examination was also restricted to 3 LLMs, specifically GPT-3.5, GPT-4, and Bard. The landscape of available language models is vast and continually evolving, and it is conceivable that different models might yield divergent results. In addition, the field lacks established guidelines for leveraging LLMs to optimize accuracy. Notwithstanding rigorous attempts to devise specific, comprehensive prompts, it remains plausible that alternative queries could generate more precise outcomes. This fact underscores the multifaceted nature of the challenge and the need for further research in this domain.

The choice of prompt plays a crucial role in determining the output generated by LLMs. During the exploratory phase of our study, various prompt versions were tested. While our study did not focus on identifying the optimal prompts, several techniques used in our prompts appeared to enhance output quality: specifying a minimum number of papers (a minimum of 9 papers); using bullet points to delineate criteria such as “type of studies,” “participants,” and “interventions”; and explicitly instructing to “exclude systematic reviews and meta-analyses.” Introducing prompts by specifying the researcher’s profession provides additional context, aligning with recommendations from LLM providers. Finally, enforcing a specific reference style format facilitated the retrieval of vital information, including authors’ names, journal titles, publication dates, and DOIs when available.

Our decision not to provide the initial PubMed results list to LLMs for assessing paper eligibility was deliberate, aimed at preserving study integrity and interpretability. While providing the list might enhance LLM accuracy, it introduces bias by guiding models toward replicating the provided set rather than autonomously identifying relevant studies. Our study design, though sacrificing some precision, ensures that LLM results reflect genuine capabilities in navigating scientific literature independently.

### Future Directions

LLMs present a highly efficient instrument that may aid academics in the drafting of research papers. However, upon analyzing the findings of this study, it becomes imperative to emphasize that the bibliographic references proposed by the AI are not intrinsically trustworthy. These citations necessitate human validation, focusing on the authors, the title, and the subject matter.

We thereby deduce that, in the context of GPT iterations, user verification is indispensable for preserving the scientific integrity and relevance of the output. A statement or a scholarly usage guideline should be prominently featured before the tool is used or should be integrated into the software itself to outline its lack of liability for any inaccuracies in the citation of papers. This is paramount as such errors could potentially mislead a considerable number of users. We also propose that the application of GPT-based chatbots for tasks such as spelling correction, proofreading, or text restructuring ought to be explicitly mentioned within the materials and methods section of academic writings.

### Conclusions

ChatGPT and Bard exhibit the capacity to generate convincingly authentic references for systematic reviews but also yield hallucinated papers in 28.6% (34/119) to 91.3% (95/104) of cases. Among the models tested, GPT-4 displayed superior performance in generating legitimate and relevant references but, like the other models, largely failed to respect the established eligibility criteria. Given their current state, LLMs such as ChatGPT and Bard should not be used as the sole or primary means for conducting systematic reviews of literature, and it is crucial that references generated by these tools undergo rigorous validation by the authors of scientific papers.

## Appendix

Multimedia Appendix 1 Search strategy.

## Abbreviations

AI: artificial intelligence
LLM: large language model
PRISMA: Preferred Reporting Items for Systematic Reviews and Meta-Analyses

## Data Availability

All papers accessed by the large language models (LLMs) were publicly available, and no proprietary or subscription-based sources were used without appropriate access rights. LLMs did not have access to any sensitive or private patient data. The data sets generated and analyzed during this study are available from the corresponding author on reasonable request.
