# Supplementary material for: Hallucination Rates and Reference Accuracy of ChatGPT and Bard for Systematic Reviews: Comparative Analysis
Source: J Med Internet Res. 2024 May 22;26:e53164. doi: 10.2196/53164 (PMC11153973; doi:10.2196/53164)
Supplement: Multimedia Appendix 1 [file jmir_v26i1e53164_app1.docx]

(("shoulder"[MeSH Terms] OR "shoulder"[All Fields] OR "shoulders"[All Fields] OR "shoulder s"[All Fields]) AND ("rotator cuff"[MeSH Terms] OR ("rotator"[All Fields] AND "cuff"[All Fields]) OR "rotator cuff"[All Fields]) AND ("random allocation"[MeSH Terms] OR ("random"[All Fields] AND "allocation"[All Fields]) OR "random allocation"[All Fields] OR "randomization"[All Fields] OR "randomized"[All Fields] OR "random"[All Fields] OR "randomisation"[All Fields] OR "randomisations"[All Fields] OR "randomise"[All Fields] OR "randomised"[All Fields] OR "randomising"[All Fields] OR "randomizations"[All Fields] OR "randomize"[All Fields] OR "randomizes"[All Fields] OR "randomizing"[All Fields] OR "randomness"[All Fields] OR "randoms"[All Fields])) AND ((systematicreview[Filter]) AND (2020:2020[pdat]))
